# Congenital infection with *Anaplasma phagocytophilum* in a calf in northern Germany

**DOI:** 10.1186/1751-0147-55-38

**Published:** 2013-05-01

**Authors:** Thomas Henniger, Pauline Henniger, Thekla Grossmann, Ottmar Distl, Martin Ganter, Friederike D von Loewenich

**Affiliations:** 1 Institute for Animal Breeding and Genetics, University of Veterinary Medicine Hannover, Bünteweg 17p, Hannover, D-30559, Germany; 2 Clinic for Swine and Small Ruminants, University of Veterinary Medicine Hannover, Bischofsholer Damm 15, Hannover, D-30173, Germany; 3 Institute of Medical Microbiology and Hygiene, University of Freiburg, Hermann-Herder-Strasse 11, Freiburg, D-79104, Germany

**Keywords:** *Anaplasma phagocytophilum*, Intrauterine infection, Tick-borne fever, Cattle, Germany

## Abstract

*Anaplasma phagocytophilum* is a Gram-negative, obligate intracellular tick-transmitted bacterium that replicates in neutrophils. It causes tick-borne fever (TBF) in sheep and cattle, but also elicits febrile disease in humans as well as in other domestic animals such as dogs, horses, and cats. Although increasingly recognized in Europe, the first laboratory-confirmed case of TBF in cattle from Germany has been published only recently. We here present the unusual case of an intrauterine transmission of *A. phagocytophilum* in a calf from northern Germany. To our knowledge, this is the first report of such an event occurring under field conditions in cattle.

## Background

*Anaplasma phagocytophilum* is a Gram-negative, obligate intracellular tick-transmitted bacterium that replicates in neutrophils [1]. It was initially recognized as the causative agent of tick-borne fever (TBF) in sheep and cattle [2,3], but also causes febrile disease in dogs [4], horses [5], cats [6], and humans [1]. As a result of taxonomic changes in 2001, *A. phagocytophilum* comprises the former species *Ehrlichia phagocytophila*, *E. equi*, and the agent of human granulocytic ehrlichiosis [7]. However, this unification has been a matter of debate, because naturally circulating *A. phagocytophilum* strains are not equally infectious for different mammalian species [8,9]. The main vector of *A. phagocytophilum* in Europe is *Ixodes ricinus*[10]. Clinical symptoms in cattle include high fever, reduced milk yield, inappetence, cough, and abortion [10]. Leukopenia, thrombocytopenia, and anemia are typical laboratory findings [11]. The diagnosis is made from peripheral blood via microscopic detection of bacterial inclusions in granulocytes, so-called morulae, or by species-specific polymerase chain reaction (PCR) [12]. In contrast, the significance of serological tests is very limited, because they do not distinguish between recent or past infections [13]. Although increasingly recognized in Europe, the first laboratory-confirmed case of TBF in cattle from Germany has been published only recently [14]. We here report an unusual case of intrauterine transmission of *A. phagocytophilum* to a calf.

## Case presentation

### Clinical signs

In June 2011, a male Holstein-Friesian calf was obtained immediately after birth from a dairy farm in Lower-Saxony for inclusion in a study investigating the pathogenesis of alloantibody-induced bovine neonatal pancyotopenia (BNP) [15]. The dam was born in January 2007 and raised on the farm. She calved for the first time in 2009 and aborted in 2010 after her second mating. Thereafter, in September 2010 the cow was artificially inseminated. From May 2011 she was grazed on pasture. With the exception of an intramammary antibiotic treatment, the cow did not receive any systemic antimicrobials.

The animal experiments investigating BNP were approved by the Animal Welfare Committee of the Niedersächsisches Landesamt für Verbraucherschutz und Lebensmittelsicherheit, Oldenburg (reference number: 33.9-42502-04-09/1799). EDTA blood and serum samples were collected from the precolostral calf and its mother. The calf was then fed with colostrum that was known to induce BNP. Since its first day of life, the calf showed elevated rectal temperatures of at least 39.8°C. In contrast to the other animals from the same experimental group that developed BNP 3 to 4 days after birth, the calf already showed typical signs of BNP such as cutaneous hemorrhages, mucosal petechial hemorrhages, and melena from its second day of life onwards. Similarly, thrombocytopenia, leukopenia, and anemia developed rapidly (Table 1). At the age of 4 days, the calf was euthanized, because it stopped drinking and showed severe deterioration in its general condition and persistent recumbency. Because the dam was asymptomatic, she was not medicated.

**Table 1:** Rectal temperatures and blood cell counts of the calf and its mother

	**Reference values calves**^**+**^	**Calf**						**Reference values cattle**^**+**^	**Dam**
**Day after birth**		**0**	**0**	**1**	**2**	**3**	**4**		**4**
**Time**		**02:00**	**19:30**	**08:30**	**08:30**	**08:30**	**11:00**		**nd**
**Rectal temperature** (°C)	38.5 - 39.5	nd	40.1	39.8	39.9	40.1	39.9	38.0 - 39.0	nd
**Hemoglobin** (g/l)	75 - 120	125	nd	97	80	59	40	80 - 120	139
**Packed cell volume** (l/l)	0.22 - 0.46	0.40	nd	0.30	0.24	0.18	0.12	0.28 - 0.39	0.40
**Red blood cell count** (x 10^12^/l)	6.0 - 10.0	9.14	nd	7.16	5.77	4.44	2.91	5.0 - 8.0	6.76
**Reticulocytes** (‰)	0 - 10	0	nd	0	0	0	3	0 - 10	1
**Thrombocytes** (x 10^9^/l)	200 - 1300	180	nd	15	14	3	23	200 - 800	323
**White blood cell count** (x 10^9^/l)	4.0 - 14.0	4.9	nd	1.5	1.7	3.2	2.7	5.0 - 10.0	10.5
**Lymphocytes** (x 10^9^/l)	2.5 - 5.5	1.81	nd	0.50	0.44	0.32	0.26	2.5 - 5.5	5.72
**Monocytes** (x 10^9^/l)	0.2 - 0.8	0.17	nd	0.03	0.00	0.00	0.00	0.2 - 0.8	0.52
**Segmented neutrophils** (x 10^9^/l)	2.5 - 4.5	2.87	nd	0.72	0.99	2.21	0.99	2.5 - 4.5	3.88
**Band neutrophils** (x 10^9^/l)	0.0 - 0.2	0.05	nd	0.24	0.27	0.67	1.17	0.0 - 0.2	0.37
**Eosinophils** (x 10^9^/l)	0.3 - 0.9	0.00	nd	0.02	0.00	0.00	0.00	0.3 - 0.9	0.00
**Neutrophils with morulae** (% of the examined 400)	0	*	nd	60**	53	47	56	0	0

^+^[16,17], nd = not determined, *not definable due to cell damage, **30% of lymphocytes and some monocytes also infected.

### Laboratory test results

**Figure 1:**
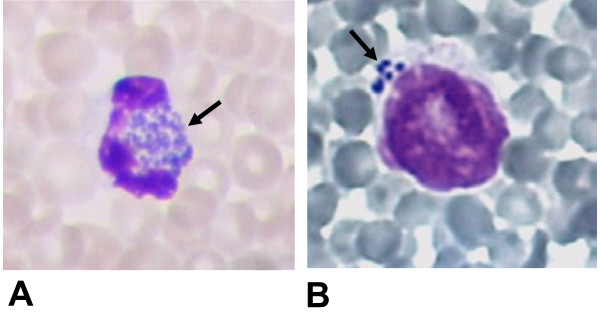
**Morulae (arrow) of *Anaplasma phagocytophilum *in Giemsa-stained blood smears of the calf.** Morulae in a neutrophil in a blood smear at 2 hours after birth. (**A**). Morulae in a lymphocyte in a blood smear at day 1 after birth (**B**). Magnification: 10 × 100.

Results of blood counts and the course of the rectal temperature of the calf are shown in Table 1. In a Giemsa-stained smear of precolostral EDTA blood, morulae of *A. phagocytophilum* were detected in neutrophil granulocytes (Figure 1A). One day after birth, morulae were not only found in 60% of the 400 examined neutrophils, but also in 30% of the lymphocytes (Figure 1B) and in some monocytes. Blood smears remained positive for *A. phagocytophilum* until the calf was euthanized. Maternal blood smears were microscopically negative.

The diagnosis was confirmed by amplification and sequencing of the 16S rRNA gene of *A. phagocytophilum* from blood samples taken on days 3 and 4 after birth as described previously [18,19]. The 16S rRNA gene sequence was identical to an *A. phagocytophilum* variant [GenBank: M73220] well-known mainly from ruminant infections. EDTA blood samples from the dam taken at parturition and 2 days postpartum contained the same 16S rRNA genotype. At day 19 postpartum, the dam was found to be negative for *A. phagocytophilum* DNA.

Serum samples from both animals were investigated by an indirect immunofluorescence test (IFT) (Focus Diagnostics, Cypress, CA, USA) using FITC-conjugated goat anti-bovine IgG (H + L) antibody (Dianova, Hamburg, Germany) as secondary antibody. Serum from the 2-day-old calf was negative for anti-*A. phagocytophilum* antibodies, whereas the cow had a titer of 1:256 (cut-off: 1:64) on days 2 and 19 post partum.

To determine whether both animals were infected by the same strain of *A. phagocytophilum*, the *ankA* gene as a further genotypic marker was amplified as described previously [20,21]. Unexpectedly, two *ankA* gene sequences belonging to two different clusters, I and IV [21] were amplified from the calf’s blood at days 3 and 4 after birth. In contrast, the dam was infected with *A. phagocytophilum* carrying only the *ankA* gene variant of cluster I in blood samples taken at parturition and 2 days postpartum. The cluster I *ankA* sequences of both animals were 100% identical. To confirm that both animals were infected with the same *A. phagocytophilum* strain, multi locus sequence typing (Winter C, Huhn C, Wolfsperger T, Wüppenhorst N, von Loewenich FD: unpublished observations) was applied and revealed the same sequence type 140 in mother and calf. An overview of the *A. phagocytophilum-*specific test results is shown in Table 2. 16S rRNA and *ankA* gene sequences are available under the following accession numbers: [GenBank: KC776917 – KC776921].

**Table 2:** **Summary of *Anaplasma phagocytophilum-*specific test results**

**Day after birth**	**Calf**	**Dam**
**0**	- morulae in blood smear	- microscopically negative
		- 16S rRNA gene identical to [GenBank: M73220]
		- detection of *ankA* cluster IV
		- sequence type 140
**1**	- morulae in blood smear	
**2**	- morulae in blood smear	- microscopically negative
	- IFT < 1:64	- 16S rRNA gene identical to [GenBank: M73220]
		- detection of *ankA* cluster IV
		- sequence type 140
		- IFT 1: 256
**3**	- morulae in blood smear	
	- 16S rRNA gene identical to [GenBank: M73220]	
	- detection of *ankA* cluster I and *ankA* cluster IV	
	- sequence type 140	
**4**	- morulae in blood smear	- microscopically negative
	- 16S rRNA gene identical to [GenBank: M73220]	
	- detection of *ankA* cluster I and *ankA* cluster IV	
	- sequence type 140	
**19**		- microscopically negative
		- 16S rRNA gene negative
		- IFT 1:256

## Discussion

Although TBF in cattle is known to cause abortion or stillbirth [10], to our knowledge a spontaneously occurring intrauterine infection of a bovine fetus with *A. phagocytophilum* has not been reported to date. In our case, evidence for an intrauterine infection comes from the fact that a significant proportion of neutrophils in the blood smear of the calf prepared 2 hours after birth were already infected. Furthermore, the calf was not tick-exposed during its short lifetime. It has been shown that oral transmission of *A. phagocytophilum* is inefficient in cattle [22], whereas it has been proven experimentally that intrauterine infection does occur [23]. That study showed absence of specific fetal antibodies in a calf delivered 17 days after the mother was experimentally infected and that the calf did not seroconvert until it was 26 days old [23]. The calf reported in the present case was likewise seronegative. Based on these circumstances, it seems most likely that it was infected *in utero*. It has been shown experimentally that adult cattle develop elevated antibody titers of > 1:320 at day 14 post infection and remain PCR-positive for *A. phagocytophilum* on average until day 22 [11]. Because the dam was PCR positive and showed an antibody titer of 1:256 at day 2 after parturition, we suggest that she had been infected 2 to 3 weeks earlier. As far as reported by the farmer the dam was clinically asymptomatic, but the symptoms that typically last for 1 week [11] might have gone unrecognized.

The molecular characterization of the *A. phagocytophilum* strain proved that mother and calf were infected with the same strain. However, two *ankA* gene variants were found reproducibly in the calf’s blood. The possibility exists that the dam initially transmitted two strains that were not distinguishable apart from their *ankA* genes and that she had already cleared one of them at the time of delivery. It might also be that the bacterial burden of one of the strains had already fallen below the detection limit. Alternatively, intra-species recombination, as has been hypothesized earlier [21], could have taken place leading to the appearance of two *ankA* gene variants in the calf’s blood.

## Conclusions

A spontaneous bovine congenital infection with *A. phagocytophilum* is reported for the first time. Intrauterine infection of cattle has previously been produced experimentally, but this study confirms that this may also occur under field conditions.

## Consent

Written informed consent was obtained from the owner for publication of this report.

## Abbreviations

BNP: Bovine neonatal pancytopenia; IFT: Indirect immunofluorescence test; PCR: Polymerase chain reaction; TBF: Tick-borne fever.

## Competing interests

The authors declare that they have no competing interests.

## Authors’ contributions

TH and PH did the clinical investigation. TG, MG, and FvL performed the laboratory tests. TH, OD, MG, and FvL prepared the manuscript. All authors have read and approved its final version.
