# Correction to: Congenital infection with *Anaplasma phagocytophilum* in a calf in northern Germany

**DOI:** 10.1186/s13028-019-0444-6

**Published:** 2019-01-30

**Authors:** Thomas Henniger, Pauline Henniger, Thekla Grossmann, Ottmar Distl, Martin Ganter, Friederike D. von Loewenich

**Affiliations:** 1 Institute for Animal Breeding and Genetics, University of Veterinary Medicine Hannover, Bünteweg 17p, 30559 Hannover, Germany; 2 Clinic for Swine and Small Ruminants, University of Veterinary Medicine Hannover, Bischofsholer Damm 15, 30173 Hannover, Germany; 3 Institute of Medical Microbiology and Hygiene, University of Freiburg, Hermann-Herder-Strasse 11, 79104 Freiburg, Germany

## Correction to: Acta Vet Scand (2013) 55:38 10.1186/1751-0147-55-38

Following publication of the original article [1], we have been notified of a typing error under the “Laboratory test results” section.

- Incorrect sentences:
  - In contrast, the dam was infected with *A. phagocytophilum* carrying only the *ankA* gene variant of **cluster I** in blood samples taken at parturition and 2 days postpartum.
  - The **cluster I** *ankA* sequences of both animals were 100% identical.
- Correct sentences:
  - In contrast, the dam was infected with *A. phagocytophilum* carrying only the *ankA* gene variant of **cluster IV** in blood samples taken at parturition and 2 days postpartum.
  - The **cluster IV** *ankA* sequences of both animals were 100% identical.
